# Brick plots: an intuitive platform for visualizing multiparametric immunophenotyped cell clusters

**DOI:** 10.1186/s12859-020-3469-y

**Published:** 2020-04-15

**Authors:** Samuel E. Norton, Julia K. H. Leman, Tiffany Khong, Andrew Spencer, Barbara Fazekas de St Groth, Helen M. McGuire, Roslyn A. Kemp

**Affiliations:** 1 Department of Microbiology and Immunology, University of Otago, Dunedin, New Zealand; 2 Myeloma Research Group, Australian Centre for Blood Diseases, Alfred Hospital-Monash University, Melbourne, VIC Australia; 3 Malignant Hematology and Stem Cell Transplantation, Alfred Hospital, Melbourne, VIC Australia; 4 Ramaciotti Facility for Human Systems Biology, The University of Sydney and Centenary Institute, Sydney, Australia; 5 Discipline of Pathology, School of Medical Sciences, Faculty of Medicine and Health, The University of Sydney, Australia; Charles Perkins Centre, University of Sydney, Sydney, Australia

**Keywords:** Mass cytometry, Analysis, T cells, NK cells, Cancer, Clinical

## Abstract

**Background:**

The advent of mass cytometry has dramatically increased the parameter limit for immunological analysis. New approaches to analysing high parameter cytometry data have been developed to ease analysis of these complex datasets. Many of these methods assign cells into population clusters based on protein expression similarity.

**Results:**

Here we introduce an additional method, termed Brick plots, to visualize these cluster phenotypes in a simplified and intuitive manner. The Brick plot method generates a two-dimensional barcode that displays the phenotype of each cluster in relation to the entire dataset. We show that Brick plots can be used to visualize complex mass cytometry data, both from fundamental research and clinical trials, as well as flow cytometry data.

**Conclusion:**

Brick plots represent a new approach to visualize complex immunological data in an intuitive manner.

## Background

The advent of mass cytometry, spectral cytometry and other high parameter protein analyses has revolutionized the way we study immune cell populations. It has become clear that it is both difficult and time consuming to observe the vast heterogeneity among immune cells using older techniques designed for low parameter data. Moreover, analyses of immune cell function can be re-examined with more modern technologies and data analysis approaches [1]. As with the development of genome sequencing, the limiting factor in understanding immune heterogeneity in health and disease, is our ability to analyse, interpret and visualize the vast quantities of data [2].

Advanced bioinformatic approaches to data analysis are relatively new to immunology. While some groups have pioneered the development of new bioinformatics methods in recent years [3–8], a large proportion of published immunological research does not use advanced analysis methods. A range of platforms and analysis tools that allow all immunologists and non-immunologists to use and understand these datasets are now available [1, 3, 4, 6, 7]. However, in our own prior research, the biggest hurdle to understanding and presenting the results of such analyses was the visualization of cluster phenotypes and the ability to clearly communicate results to a broad research audience.

Here we propose a new method for visualizing immune cell phenotypes downstream of cluster analyses. We generate a two-dimensional barcode that describes the phenotype of a given immune cell population. This barcode is called a Brick plot and comprises “bricks” that each represent a marker, with the position of the brick in two-dimensional space reflecting its correlation to all other markers. We believe these Brick plots represent an intuitive approach to visualizing complex immune cell populations.

## Results

### A two-dimensional barcode of cluster phenotype

**Fig. 1:**
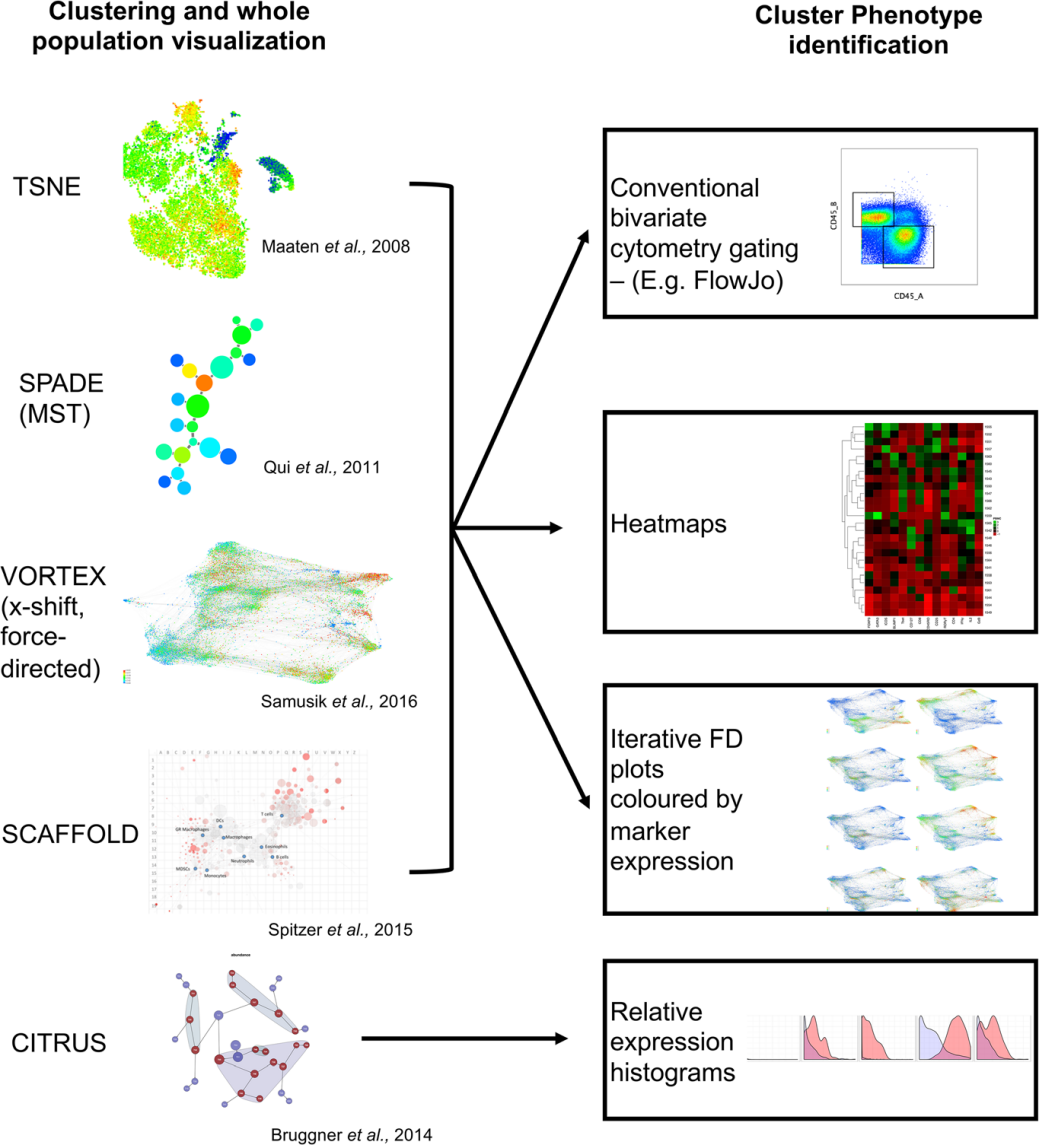
Current approaches to analyse mass cytometry data. (Left) 5 examples of commonly used approaches for visualising mass cytometry data. tSNE or viSNE, SPADE or other minimum spanning tree (MST) approaches, x-Shift clustering in the VorteX application and subsequent force-directed layout, SCAFFoLD and CITRUS. (Right) Downstream approaches to determining cluster/subset phenotype. Conventional cytometry analysis, heatmaps, iterative plotting of visualisation coloured by a single marker and relative expression histograms. Most visualisation techniques allow for downstream processing using any of the listed techniques, CITRUS is more restricted to built-in outputs

The principal aim of many high parameter analyses is to reduce complex data to a form that is readily interpretable by the analyst. This can be achieved with the use of clustering and dimensionality reduction techniques. Figure 1 provides a summary of five commonly used methods for visualizing mass cytometry data, together with four approaches used to illustrate the phenotype of clusters/populations defined by these methods. However, these four approaches are not always easily interpretable. Furthermore, these approaches often require considerable time and effort on the part of the researcher. We aimed to produce a visualization technique that resulted in a rapidly interpretable phenotypic readout for each cluster, regardless of the source of the cluster.

**Fig. 2:**
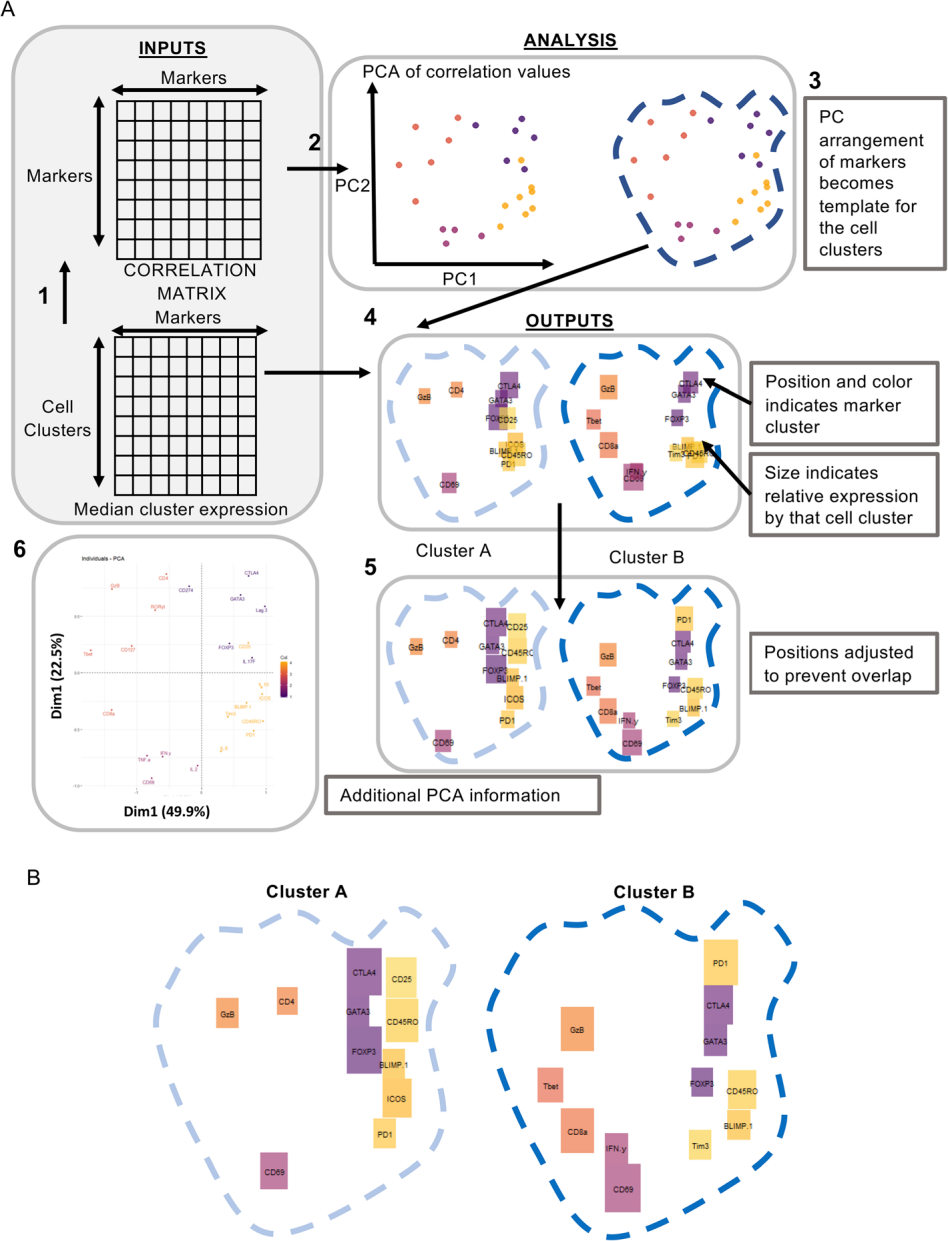
Generation of Brick plots. **a** Schematic of data processing to generate Brick plots. Brick plots require an input dataset comprising markers (columns) and samples/clusters (rows). (1) A correlation matrix is generated from the dataset and a principal component analysis performed on the matrix (2). Each marker is plotted based on its PC1, PC2 co-ordinate defining the location of each marker in the plot (3). Locations are coloured based on K-means cluster-assignation. Expression values for each marker in each sample are used to define the size of boxes to be plotted (4). Overlapping bricks are adjusted to remove overlap (5), initial locations are plotted with PC1/2% variance shown (6) **b** Example Brick plots for T cell populations generated from CRC patient cohort 1 tissue samples (pooled) acquired by mass cytometry (Additional file 5). Cluster A represents a CD4 T cell population. Cluster B a CD8 T cell population. Brick size indicates relative expression of the labelled marker, colour indicates marker cluster assignation, based on broad categories – orange represents T cell lineage and cytotoxic functions (CD4, CD8, Tbet, GzB); pink represents antigen exposure and effector function (IFN-γ, CD69); purple represents regulatory transcription factors and proteins (FOXP3, CTLA4, GATA3), and yellow represents activation and inhibitory receptors (CD25, CD45RO, BLIMP-1, ICOS, PD-1, Tim-3).

We propose a two-dimensional barcode for each cluster phenotype composed of bricks that represent each marker expressed in that cluster. We have termed these “Brick plots” (Fig. 2). For each defined cell cluster, bricks are located based on the co-expression correlation between their respective markers - bricks that represent markers that are commonly co-expressed in the given dataset are closely positioned, for example CTLA-4 and GATA3 in Fig. 2b Cluster B (top right). The relative size of each brick indicates the relative expression of that marker in that cluster compared to other clusters – a large brick indicates high relative expression and no brick indicates no, or low, relative expression. The result is an immediately interpretable visualization of the cluster phenotype (Fig. 2).

The Brick plot code can be found at https://github.com/NortonS1/BrickPlots. Users of this package are expected to have familiarity of how to install packages in R, although instructions on how to do this are included. The first step is to designate marker-brick position based on co-expression. A correlation matrix is produced using median marker expression within each cluster. This provides a value from − 1 to 1 indicating the relative co-expression of each marker to each other marker within the dataset (Fig. 2a.1). Next a principal component analysis (PCA) is performed on the correlation matrix. Demarking the greatest variance as scalar projections, principal component (PC)1 and PC2 are used as the axes for determining brick-marker location (Fig. 2a.2). In addition to the PCA, an iterative K-means clustering step is performed to group the clusters. This is performed with 30 K values from one to the number of parameters in the dataset. Elbow point validation is used to decide the most accurate K value (tested between number1 and number2). Marker-brick positions are then grouped (by color) based on K-means group assignation (Fig. 2a.2). This approach not only groups markers for ease of interpretation, but can also provide additional information regarding co-expression of functional markers.

The second step is to place bricks at the previously defined locations. For this, the area of each given brick is a scaled representation of the arcsinh transformed expression value for that given marker in that cluster. This results in large bricks for highly expressed markers and small bricks for low, but still positive expression. Markers within a cluster with arcsinh transformed values below one for that marker are zeroed – resulting in no brick, which reduces noise and improves interpretability (Fig. 2a.4, b). We selected a value of one based on background signal reaching a maximum of one in most markers, however, this value can be easily altered by the user to suit any given background threshold (Additional file 4). Arcsinh transformation is utilized as a convention with mass cytometry data, deemed suitable for transforming the measured ion counts as it resembles a log transformation in the upper range while retaining linearity for low counts. The range of the ion counts where linearity is retained can be adjusted by dividing counts by a user-defined factor before ArcSinh transformation, thus emphasizing the signal in the lower end of the spectrum. For CyTOF data, a co-factor around 5 is typically used—that is, all counts are divided by 5 to deemphasize noise around 0.

This approach to brick localization often resulted in considerable brick overlap making interpretation of cluster phenotype challenging. As such, an additional step was added to detect for brick overlap and adjust the location by placing the new brick adjacent to the existing overlapped brick (Fig. 2a.5).

Figure 2b depicts Brick plots for two clusters identified within mass cytometry data generated from Cohort 1 CRC tissue samples using VorteX. In Fig. 2b, cluster B has a large IFN-γ brick (bottom left), indicating high relative IFN-γ expression. The IFN-γ brick is absent in cluster A indicating very low or no IFN-γ expression. Cluster B, however, has some T-bet expression, although this expression is not as high as other populations in the analysis, indicated by a small brick.

Clusters A and B are a subset of the full complement of populations generated from Cohort 1 and shown in Additional file 1 as a heatmap, with a direct comparison to a single cluster expressed as a Minimum spanning tree (MST) plot or a Brick plot. Comparison of clusters A and B illustrated as Brick plots (Fig. 2b) with the heatmap (Additional file 1) revealed that all markers depicted in either of the two Brick plots (cluster 20,313 is A, cluster 20,315 is B) appear as green (high expression) boxes in the heatmap, and no red boxes (low/no expression) from the heatmap are shown on the Brick plots (Fig. 2b, Additional file 1).

**Fig. 3:**
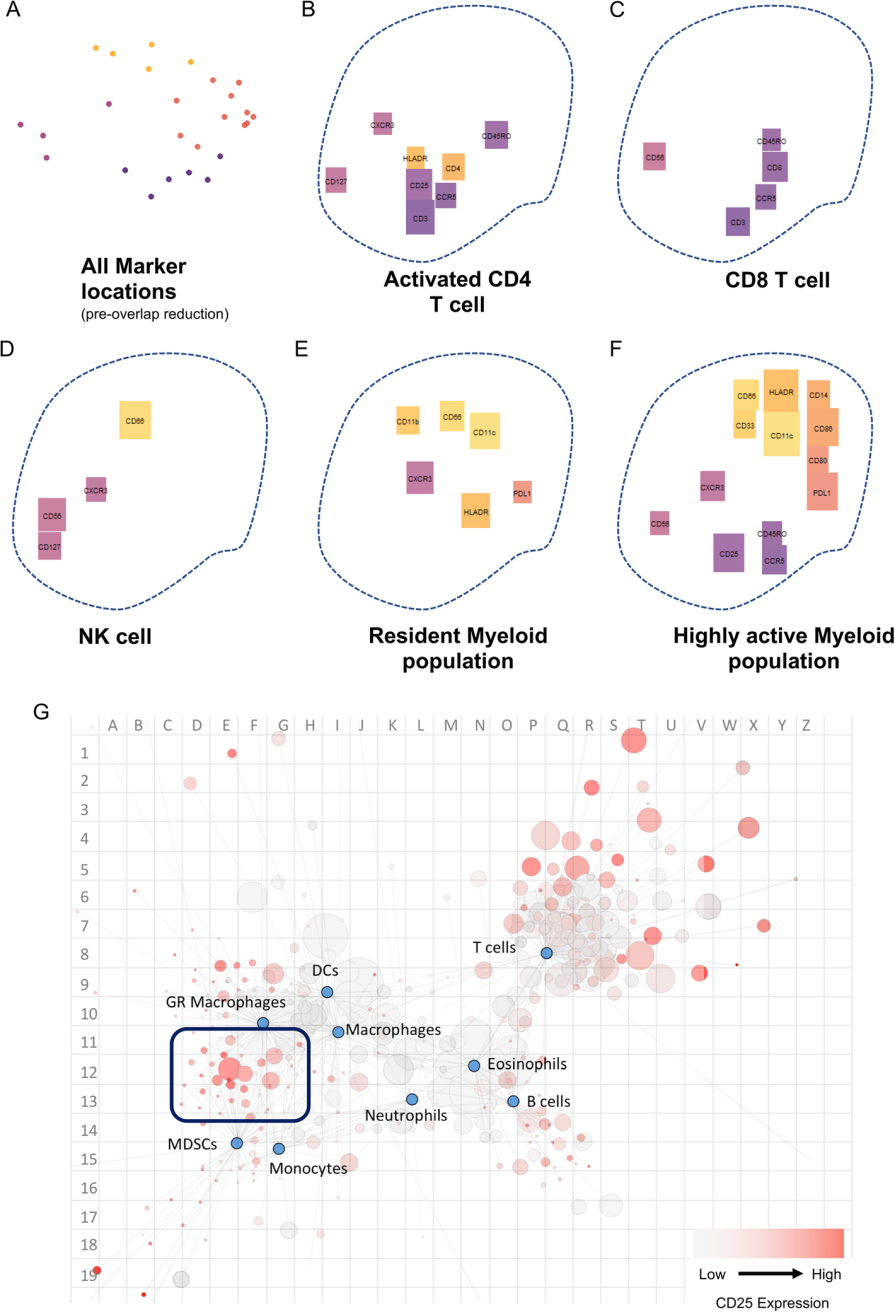
Brick plots enable visualization of immune cell populations. Data used was acquired by mass cytometry from CRC patient cohort 2 (pooled, Additional file 6). **a-f** Brick plots generated downstream of x-shift clustering in VorteX. (A) all marker locations defined by PCA and coloured by K-means cluster assignation – pink represents markers associated with T cells, orange represents markers associated with resident myeloid cells, light purple represents markers associated with NK cells and dark purple represents markers associated with activated myeloid cells. **b-f** Brick size indicates relative expression of the labelled marker, colour indicates marker cluster assignation. **g** SCAFFoLD plot generated using the same dataset, with grid reference overlay added post-analysis. Blue dots indicate predefined landmark nodes, grey (low) to red (high) dots indicate relative CD25 expression. The size of each node indicates relative population size. The purple box outlines the CD25hi myeloid populations depicted in (**f**)

We used mass cytometry data acquired from human colorectal tumour and non-tumour bowel (NTB) samples as a test dataset to validate the use of Brick plots to identify canonical and novel immune cell populations. This data had previously been assessed using SCAFFoLD [1]. Here we used x-shift in the VorteX application to define cell clusters and generated Brick plots of five out of ten of the major clusters descried by VorteX [7] (Fig. 3).

The Brick plots provided a clear visualization of canonical immune cell populations but also revealed unexpected populations. Markers were grouped into four clusters, indicated by color, by K-means clustering (Fig. 3a). T cell markers were predominantly in pink, resident myeloid cell markers in orange, natural killer (NK) cells in light purple and activated myeloid cell markers in dark purple.

We identified activated CD4 T cells (Fig. 3b), CD8 T cells (Fig. 3c), an NK cell population (Fig. 3d), a resident myeloid population [9] (Fig. 3e), and an additional myeloid population with high expression of markers including CD25 (bottom centre, Fig. 3f). Importantly, co-expression or mutually exclusive expression was consistent with previous reports. By grouping the bricks based on co-expression, it was easy to identify T cell populations (Fig. 3b, c) by their pink brick predominance versus myeloid cell populations (Fig. 3e, f) with dark purple and orange brick dominance.

The Brick plot in Fig. 3b shows an activated CD4 T cell population. The population has large CD3 and CD4 bricks (centre) indicating a CD4 T cell population. In addition, this population has large CD45RO (right) and intermediate CD25 (centre) bricks indicating an activated CD4 T cell phenotype. In comparison, the population depicted in Fig. 3c has a large CD8 brick and intermediate CD3 (centre), corresponding to CD8 T cells. And, while still expressing CD45RO, the relative size of this brick compared to Fig. 3b indicates lower expression of this markers in this population. The presence of a small CD56 brick (left) in Fig. 3c is likely the result of the general low CD56 expression within the assessed dataset. Genuine CD56 expression is depicted in the NK cell population in Fig. 3d has a large CD56 brick compared to Fig. 3c.

The list of immunological markers included in this panel was not targeted towards identifying NK cell population. The NK cell population in Fig. 3d had high expression of CD56, CD66 and CD127 – the interpretation of these data suggests a possible NK/innate lymphoid cell (ILC)3 population [10, 11] or even a T cell population [12]. This identification of an NK cell population highlights the capability of Brick plots to correctly reveal populations that were not explicitly targeted without creating noise from a large number of negative markers.

### Brick plots provide an intuitive visualization of unpredicted populations

The presence of a CD25^hi^ macrophage-like population in the tumour tissue was a key finding from the previously unpublished analysis of this dataset in SCAFFoLD (Fig. 3g). Broad lineage-level populations were defined prior to the SCAFFoLD analysis using conventional cytometry approaches such as bivariate gating. These populations formed the “scaffold” of blue nodes in the SCAFFoLD plot. Grey to red nodes represent raw data. The size of a node indicates the relative abundance of that exact phenotype. The proximity of a raw node to a landmark indicates its similarity to the initially defined lineage. In this particular plot, the grey to red axis indicates relative low to high CD25 expression. The purple box at co-ordinates C-G, 11–13 (Fig. 3g) highlights a population of CD25 high cells that are situated closely to myeloid lineages, particularly macrophage subsets - defined by expression of CD64, CD33, CD11b, CD14, HLA-DR at various levels. This population is more clearly described as the Brick plot, Fig. 3f. The large relative size of all dark purple and orange bricks, that represent common myeloid markers, in addition to a large CD25 brick (bottom) immediately reveals a CD25^Hi^ myeloid population. This population also expresses markers more commonly associated with T cell subsets, such as CD45RO and CCR5 (bottom right). However, the population in Fig. 3f does not express CD3, and further, it is possible for these markers to be expressed in myeloid populations [13–15]. We also verified the existence of this CD25 myeloid population by manual gating (Additional file 3). To ascertain the same information displayed in the Brick plot using SCAFFoLD, the researcher would have to cycle through every available marker in the same manner as CD25 is portrayed in Fig. 3g, and manually list marker expression.

### Brick plots are also useful for low parameter flow cytometry data

**Fig. 4:**
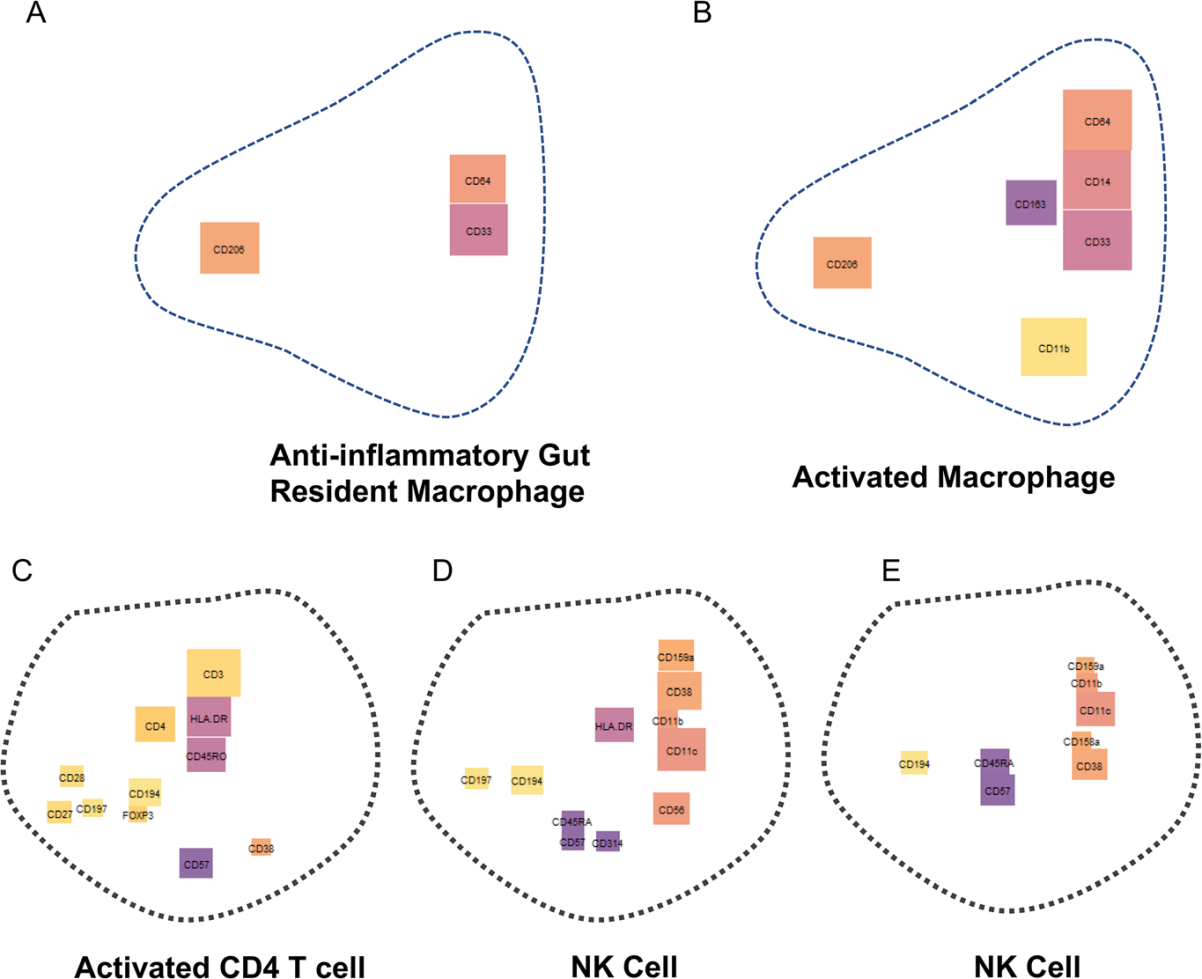
Brick plots can be used to visualise data acquired by flow cytometry and data from clinical trials. Brick plots generated downstream of x-shift clustering in VORTEX. Brick size indicates relative expression of the labelled marker, colour indicates cluster assignation. **a-b** Data used was acquired by flow cytometry from CRC patient cohort 3 (pooled, Additional file 7). **c-e** Data used was acquired by mass cytometry from MM patient cohort 4 (pooled, Additional file 8)

The increase in available parameters was the main justification for the development of Brick plots. However, low parameter analyses may also benefit from alternative visualization methods to define population phenotypes. In our previous work, we used flow cytometry to define macrophage populations in tumour and non-tumour bowel from people with colorectal cancer [9]. Considerable effort was made to design a gating strategy using conventional bivariate gating methods in order to define these macrophage populations. We have subsequently used cluster analyses and Brick plots to re-assess these populations (Fig. 4a, b). Our results mirrored our initial findings [9]; we defined a gut resident macrophage population (Fig. 4a) and a more activated macrophage population (Fig. 4b). These Brick plots thus describe the same phenotypes as conventional methods, but in a simpler format, and via a more streamlined pipeline.

Many clinical trials are interested in immune cell populations as blood based biomarkers. We used Brick plots to assess a mass cytometry dataset generated from a recent pomalidomide/dexamethasone versus pomalidomide alone treatment trial in patients with multiple myeloma (Fig. 4c-e). All samples analyzed were from treated patients. The panel was targeted towards the identification of all PBMC immune subsets. Using Brick plots, we readily identified T cell populations (Fig. 4c), multiple NK cell populations (Fig. 4d and e), and B cell and myeloid derived subsets (data not shown).

## Discussion

We aimed to streamline the analysis process for mass cytometry data analysis. Current approaches are excellent at reducing the high parameter data to a comprehensible level. However, understanding the phenotypes of each of the populations defined using these approaches is still a laborious process. Brick plots represent a new approach to visualizing the phenotype of an immune cell population defined by cluster analyses. Brick plots are based on a two-step process to produce an intuitive visualization of cluster phenotype using a two-dimensional barcode of bricks representing marker expression.

t-Distributed Stochastic Neighbor Embedding (tSNE) and Spanning-tree Progression Analysis of Density-normalized Events (SPADE) (or other MST variants) are commonly used to analyse mass cytometry data. Korin et al.*,* 2018 [16] described methods for using both tSNE (viSNE) and SPADE to analyse immune cell populations in the brain. Using this approach, the authors precisely defined a broad range of immune cells in the brain and could delineate them from non-immune cell populations based on CD45 expression. The authors state that the SPADE plot generated is an accumulation of several individually assessed SPADE plots which highlights the convoluted nature of defining population phenotypes using this approach. The use of Brick plots as a final step to their existing analysis would greatly streamline population phenotyping and provide an alternate visualization method.

Horowitz et al.*,* 2013 [17] used mass cytometry to assess human NK cell receptor repertoire and revealed a high degree of diversity within NK cell populations in human peripheral blood. Their analysis approach included both SPADE plots and heatmaps. The authors also implemented a one-dimensional barcode system for defining NK cell subsets based on marker expression. Brick plots could have been used in this regard to provide a more informative phenotypic description of the individual NK cell populations. Horowitz et al.*,* 2013 [17] also highlighted the complexity of presenting and describing phenotypes using multiple SPADE plots – an issue that arises with any MST or force-directed layout. An individual plot must be presented for each assessed marker, whereas all markers are displayed on a single plot with Brick plots. The addition of Brick plots to some analyses would allow clearer representation of key findings by reducing complexity due to noise.

Heatmaps are the universal tool, across many scientific disciplines, for displaying complex descriptive data. Heatmaps use colored boxes in a grid to represent relative high or low values for a given variable (column) in a given data point (row). In biology, this representation usually equates to a compact readout showing relative expression of every assessed variable (gene, protein or other) in every biological sample. For the remainder of this discussion, heatmaps will be referred to in the context of biological use. Broad patterns can be easily identified in a heatmap, but subtle population changes are not immediately recognisable. In this regard Brick plots provide a complementary alternative; providing information on the phenotype of a specific cell type, rather than a summary of all available data. In a Brick plot, in contrast to a heatmap, only the relevant markers are displayed, thus reducing noise and aiding in immediate interpretability. Heatmaps often include hierarchical clustering of variables and/or samples. This clustering allows for broad pattern recognition and also gives an indication of marker co-expression. Brick plots use K-means clustering and marker location designation by PCA to display marker co-expression. Bottcher et al used mass cytometry to study human microglial and mononuclear cell subsets [18]. The data presented in a heatmap clearly identifies clusters of cells based on groups of phenotypic markers and separates the sources of the cell types. Brick plots, used for these data, could more easily compare the T cell populations from peripheral blood mononuclear cells (PBMCs) versus cerebrospinal fluid (CSF) to identify differences in expression of common markers (such as CD3 (higher on PBMCs)) and differentially expressed markers (such as PDL-1(only on CSF cells) and CD45 (only on PBMCs)).

Mass cytometry analysis was the specific focus for the development of Brick plots. We wanted to develop a tool that was intuitive to immunologists without a strong bioinformatic background and thereby increase the accessibility of high parameter analyses. Moreover, it was important that Brick plots could be used downstream of any of the existing approaches to analysing mass cytometry data [1, 4, 6, 7]. Brick plots are not meant as a replacement for these techniques, but as an addition, to improve interpretability and provide an alternate method of presenting data. Brick plots can be generated from any dataset that produces a table of samples (clusters, populations) against markers (protein, gene or other). We produced Brick plots from mass cytometry data generated from a clinical trial of multiple myeloma treatment options. We showed that Brick plots can be used to make complex immunological populations more readily understandable by clinicians, who require rapid data interpretation to generate useful correlations with treatment efficacy. A shift towards a standard of high parameter analyses in clinical trials could be made easier by visualization approaches like Brick plots.

While we provide illustration of the use of Brick plots for display of both mass cytometry and flow cytometry data, this approach would be just as valid for describing gene expression data. For gene data, the localization of commonly co-expressed (or not co-expressed) markers within the Brick plot could provide a useful visualization to determine patterns of expression in different samples or treatment groups. With an even broader scope, Brick plots could be used to define different microbial communities. For this, each brick would represent a different species or sub-species and the size of each brick the relative abundance of that species. For studies of the microbiota, this could provide a useful tool for describing changes in different disease states, or after dietary alterations. Vich Vila et al.*,* 2018 [19] used shotgun meta-genomic sequencing to define differences in the microbiota of patients with various forms of inflammatory bowel diseases (IBD). They produced a branching visualization to describe differences in subspecies abundance between three groups of patients. Brick plots could instead have been used to provide a concise summary of the different microbial compositions; each brick would represent a species and its size the relative abundance. Changes in brick size between the three plots would highlight differences. Moreover, co-localization of species within the Brick plot could help to identify patterns of co-colonization.

Brick plots are more immediately interpretable than other available visualization techniques largely due to the removal of irrelevant markers and the grouping of commonly co-expressed markers. A single Brick plot is generated for each cluster. Brick plots were designed in this manner to expedite the phenotypic description of a given cluster. However, in some instances, understanding broad changes with respect to a given marker across all samples is a more important output. Brick plots are not well suited to this type of analysis - existing approaches, including those described in Fig. 1, would be more appropriate. Spitzer et al*,* 2015 [1] used SCAFFoLD to describe changes in PBMC immune cell composition within normal circadian rhythm. The authors were primarily interested in broad changes to the immune “landscape” as a whole, making specific population phenotyping unnecessary. A further limitation to Brick plots is the issue of excessive brick overlap when too many markers correlate to a high degree, and the need to manually move brick labels for clarity. This reduces interpretability considerably and so methods may need to be implemented within Brick plots to detect and resolve these overlaps.

## Conclusion

Brick plots represent a new and novel approach to visualizing cluster phenotypes generated from highly parametric datasets. This approach was developed to streamline the process of describing populations defined by existing analysis approaches. While Brick plots were designed for use with mass cytometry data, they are also well suited to other data types including those outside of immunology. Most importantly, Brick plots provide a more intuitive approach to defining population phenotypes that should prove accessible to users with a wider range of skillsets than currently available methods.

## Methods

### Patients

Tissue samples for Cohorts 1, 2 and 3 (Additional files 5, 6 and 7) were obtained from patients undergoing elective surgery for colorectal cancer (CRC) at Dunedin Hospital. The study was approved by the Health and Disability Ethics Committee (#14/NTA/33) and all patients gave written informed consent prior to inclusion in the study in accordance with the Treaty of Helsinki. Specimens were dissected by a pathologist.

For Cohort 4 (Additional file 8), peripheral blood (PB, 10 mL) from multiple myeloma (MM) patients was obtained following written informed consent as per the Alfred Hospital Human Ethics Committee-approved protocol. ALLG MM14, Universal Trial Number U1111–1126-2829 was approved by the Alfred Office of Ethics and Research Governance. (#546/12) Trial public title*: A prospective randomized Phase II study of single agent pomalidomide maintenance* versus *combination pomalidomide and low dose dexamethasone maintenance following induction with the combination of pomalidomide and low dose dexamethasone in patients with relapsed and refractory myeloma previously treated with lenalidomide.*

### Brick plots code

All code for Brick plots was written in R using the R Studio IDE. Fully commented code and instructions for new users can be found at (https://github.com/NortonS1/BrickPlots). Test files have been provided.

### VorteX and SCAFFoLD cluster analyses

Analyses were performed using the validated SCAFFoLD [1] and VorteX [7] packages as per the developers’ instructions. Pre-processing of data was performed in FlowJo (version X.0.7, Tree Star), and some basic reorganization of data was performed in R.

Specific settings in both VorteX and SCAFFoLD were dependent on the input samples from each cohort. Default settings were used for all options other than marker selection.

### Minimum spanning tree and Heatmap analyses

Heatmaps were produced using the standard heatmap package included in base R. Minimum Spanning Trees were produced in the VorteX package using the MST visualisation to retain the same cluster identities.

### Tissue and PB immune cell isolation

CRC tissue samples were processed as described [20, 21]. Briefly, samples were collected and digested prior to mechanical dissociation. Samples were filtered to remove additional tissue debris. For mass cytometry, samples were frozen in liquid nitrogen.

PB samples were subjected to Ficoll isolation of mononuclear cells. Red blood cells were removed by red blood cell (RBC) lysis buffer (Sigma Aldrich, St. Louis, MO). Cells were pelleted then frozen in liquid nitrogen in 10% dimethylsulfoxide (DMSO; Sigma Aldrich)/fetal calf serum (FCS;Life Technologies, Carlsbad, CA).

### Mass cytometry and flow cytometry acquisition

Cryopresereved cells were transported to the mass cytometry facility at the Ramaciotti Centre for Human Systems Biology, University of Sydney, Australia, for acquisition on a Helios upgraded CyTOF2 (Fluidigm, South San Francisco, CA, USA). For flow cytometry, samples were prepared as for mass cytometry, but instead acquired immediately on an LSR-FORTESSA (Becton Dickinson, Franklin Lakes, NJ) at the University of Otago flow cytometry facility, Dunedin, New Zealand.

For the CRC cohorts, mass cytometry samples and flow cytometry samples were incubated with antibodies and processed as previously described [20, 21]. For mass cytometry, all reagents were provided by the Ramaciotti Centre for Human Systems Biology, at the University of Sydney. Samples were incubated with cisplatin (to identify viability), blocked with Fc Receptor antibody and incubated with a metal tagged antibody master mix (Additional file 5).

For the MM cohort, 24 h prior to incubation with antibodies, cells were thawed and incubated overnight at 37 °C in complete RPMI 1640 media (Thermofisher) consisting of 10% FCS. Cells were washed in CyTOFACS buffer (PBS (Life Technologies), 0.1%BSA (Sigma Aldrich), 2 mM EDTA (Sigma Aldrich),0.05% sodium azide (Sigma Aldrich)) and barcoded using Cell-ID 20-Plex Pd Barcoding Kit (Fluidigm) as per manufacturer’s protocol. Post bar coding, cells were incubated with surface antibodies followed by intracellular antibodies using Permeabilization Buffer (eBioscience, San Diego, CA). Dead cells were identified by cisplatin. Samples were resuspended in mQ-water and filtered through a 70 μm mesh before analysis.

For flow cytometry, samples were incubated with a live/dead viability dye and incubated with fluorescently labelled antibodies [21] (Additional file 6).

## Supplementary information

**Additional file 1.** Heatmap. MST and Brick plot depicting cluster phenotypes for Cohort 1. T cell populations generated from colorectal cancer patient Cohort 1 tissue samples (pooled) acquired by mass cytometry (Additional file 5). (A) Relative expression of markers across all clusters indicated from red (low) to green (high); cluster 20,311 highlighted for comparison in (B-C). From Fig. 2b, Cluster 20,313 is cluster A; Cluster 20,315 is cluster B. (B) MST plot of the T cell populations shown in A, colored for FOXP3; cluster 20,311 highlighted; one of 34 possible SPADE plots that could be generated (see Additional file 2). (C). Brick plot of cluster 20,311, demonstrating expression of all phenotypic markers.

**Additional file 2.** MST plots of all clusters. T cell populations generated from colorectal cancer patient Cohort 1 tissue samples (pooled) acquired by mass cytometry (Additional file 5). All clusters shown in Additional file 1 shown as MST plots.

**Additional file 3.** Manually gated CD25^+^ myeloid cells. Data used was acquired by mass cytometry from CRC patient cohort 2 (pooled, Additional file 6). (Top) pooled cells from non-tumour bowel tissue, (Bottom) pooled cells from CRC tumour tissue. Cells shown were pre-gated on cisplatin^−^, DNA^+^. Cells were gated on CD3^−^, followed by CD64^+^ to define a broad myeloid population. CD25 expression was assessed on these myeloid cells.

**Additional file 4.** Cut off values. A histogram of CD45RO as an example of a cut off value. The cut off value in this instance is similar to a conventional gating strategy used for traditional flow cytometry analysis. This means that the zero value is decided based on the biological data, which will vary for each experiment and by each user depending on the research question asked. This process, while guided by controls, is somewhat subjective and requires learned expertise and a knowledge of the specific dataset. Setting this threshold for the Brick plots package usually requires assessing the raw data to determine the maximum background signal.

**Additional file 5. **Mass Cytometry Antibody Panel 1. Mass cytometry panel to assess colorectal cancer tissue samples (Cohort 1; *n* = 20).

**Additional file 6. **Mass Cytometry Antibody Panel 2. Mass cytometry panel to assess colorectal cancer tissue samples (Cohort 2; *n* = 3).

**Additional file 7. **Flow Cytometry Antibody Panel 1. Mass cytometry panel to assess colorectal cancer tissue samples (Cohort 3; *n* = 11).

**Additional file 8. **Mass Cytometry Antibody Panel 3. Mass cytometry panel to assess blood samples from multiple myeloma patients (Cohort 4; *n* = 161).

## Data Availability

The datasets generated and/or analysed during the current study are not publicly available due to patient confidentiality but are available from the corresponding author on reasonable request.

## Abbreviations

CD-: Cluster of differentiation
CRC: Colorectal cancer
CTLA-4: Cytotoxic T lymphocyte antigen − 4
CyTOF: Cytometry by time of flight
FCS: Foetal calf serum
IBD: Inflammatory bowel diseases
ILC: Innate lymphoid cell
MM: Multiple myeloma
MST: Minimum spanning tree
NK: Natural killer
NTB: Non-tumour bowel
PBMC: Peripheral blood mononuclear cells
PCA: Principal component analysis
RBC: Red blood cell
SCAFFoLD: Single-Cell Analysis by Fixed Force- and Landmark-Directed
SPADE: Spanning-tree Progression Analysis of Density-normalised Events
Tbet: T-box protein expressed in T cells
tSNE: T-Distributed Stochastic Neighbour Embedding
viSNE: T-Distributed Stochastic Neighbour Embedding –based visualisation
