# Supplementary material for: Brick plots: an intuitive platform for visualizing multiparametric immunophenotyped cell clusters
Source: BMC Bioinformatics. 2020 Apr 15;21:145. doi: 10.1186/s12859-020-3469-y (PMC7158154; doi:10.1186/s12859-020-3469-y)
Supplement: Supplementary file 5 — Additional file 5. Mass Cytometry Antibody Panel 1. Mass cytometry panel to assess colorectal cancer tissue samples (Cohort 1; n = 20). [file 12859_2020_3469_MOESM5_ESM.docx]

**Additional File 5.** Mass cytometry panel to assess colorectal cancer tissue samples (Cohort 1; n=20)

| Antibody | Conjugate | Metal |
| --- | --- | --- |
| CD11c | 127In | Indium |
| TNF-α | 139La | Lanthanum |
| CD19 | 141Pr | Praseodymium |
| CD64 | 142Nd | Neodymium |
| CD69 | 143Nd | Neodymium |
| CD8 | 144Nd | Neodymium |
| CD1c | 145Nd | Neodymium |
| CD45RO | 146Nd | Neodymium |
| CD16 | 147Sm | Samarium |
| TIM3 | 148Nd | Neodymium |
| IL-6 | 149Sm | Samarium |
| ICOS | 150Nd | Neodymium |
| CD66B | 151Eu | Europium |
| BLIMP-1 | 152Sm | Samarium |
| CD3 | 153Eu | Europium |
| RORγT | 154Sm | Samarium |
| LAG-3 | 155Gd | Gadolinium |
| IL-2 | 156Gd | Gadolinium |
| T-bet | 158Gd | Gadolinium |
| PDL-1 | 159Tb | Terbium |
| FoxP3 | 160Gd | Gadolinium |
| GATA 3 | 162Dy | Dysprosium |
| IL-10 | 163Dy | Dysprosium |
| IL-17F | 164Dy | Dysprosium |
| CD33 | 165Ho | Holmium |
| IFN-γ | 166Er | Erbium |
| CD25 | 167Er | Erbium |
| CTLA4 | 168Er | Erbium |
| GranzymeB | 169Tm | Thulium |
| CD4 | 170Er | Erbium |
| PD-1 | 171Yb | Ytterbium |
| CD127 | 172Yb | Ytterbium |
| CD11b | 173Yb | Ytterbium |
