# Supplementary material for: Brick plots: an intuitive platform for visualizing multiparametric immunophenotyped cell clusters
Source: BMC Bioinformatics. 2020 Apr 15;21:145. doi: 10.1186/s12859-020-3469-y (PMC7158154; doi:10.1186/s12859-020-3469-y)
Supplement: Supplementary file 6 — Additional file 6. Mass Cytometry Antibody Panel 2. Mass cytometry panel to assess colorectal cancer tissue samples (Cohort 2; n = 3). [file 12859_2020_3469_MOESM6_ESM.docx]

**Additional File 6.** Mass cytometry panel to assess colorectal cancer tissue samples (Cohort 2; n=3)

| Antibody | Conjugate | Metal |
| --- | --- | --- |
| CD19 | 142Nd | Neodymium |
| CD64 | 143Nd | Neodymium |
| CCR5 | 144Nd | Neodymium |
| CD4 | 145Nd | Neodymium |
| CD8 | 146Nd | Neodymium |
| CD16 | 148Nd | Neodymium |
| CD66 | 149Sm | Samarium |
| CD45RO | 152Sm | Samarium |
| CD163 | 154Sm | Samarium |
| CD86 | 156Gd | Gadolinium |
| CD33 | 158Gd | Gadolinium |
| CD11c | 159Tb | Terbium |
| CD14 | 160Gd | Gadolinium |
| PDL1 | 161Dy | Dysprosium |
| CD80 | 162Dy | Dysprosium |
| CXCR3 | 163Dy | Dysprosium |
| CX3CR1 | 164Dy | Dysprosium |
| CD127 | 165Ho | Holmium |
| CD34 | 166Er | Erbium |
| CD206 | 168Er | Erbium |
| CD25 | 169Tm | Thulium |
| CD3 | 170Er | Erbium |
| HLA-DR | 174Yb | Ytterbium |
| PD1 | 175Lu | Lutetium |
| CD56 | 176Lu | Lutetium |
| CD11b | 209Bi | Bismuth |
