# Supplementary material for: Brick plots: an intuitive platform for visualizing multiparametric immunophenotyped cell clusters
Source: BMC Bioinformatics. 2020 Apr 15;21:145. doi: 10.1186/s12859-020-3469-y (PMC7158154; doi:10.1186/s12859-020-3469-y)
Supplement: Supplementary file 7 — Additional file 7. Flow Cytometry Antibody Panel 1. Mass cytometry panel to assess colorectal cancer tissue samples (Cohort 3; n = 11). [file 12859_2020_3469_MOESM7_ESM.docx]

**Additional File 7.** Flow cytometry panel to assess colorectal cancer tissue samples (Cohort 3; n=11)

| Marker | Fluorochrome | Supplier | Clone |
| --- | --- | --- | --- |
| CD45 | BV605 | BioLegend | H130 |
| CD64 | PE | BioLegend | 10.1 |
| CD11b | PE-Cy7 | BioLegend | ICRF44 |
| CD14 | BV421 | BioLegend | M5E2 |
| CD33 | PerCP/Cy5.5 | BioLegend | WM53 |
| CD206 | APC-Cy7 | BioLegend | 15-2 |
| CD163 | APC | BioLegend | GHI/61 |
| Live/Dead | PE-Texas Red | Invitrogen | N/A |
