# Supplementary material for: Brick plots: an intuitive platform for visualizing multiparametric immunophenotyped cell clusters
Source: BMC Bioinformatics. 2020 Apr 15;21:145. doi: 10.1186/s12859-020-3469-y (PMC7158154; doi:10.1186/s12859-020-3469-y)
Supplement: Supplementary file 8 — Additional file 8. Mass Cytometry Antibody Panel 3. Mass cytometry panel to assess blood samples from multiple myeloma patients (Cohort 4; n = 161). [file 12859_2020_3469_MOESM8_ESM.docx]

**Additional File 8.** Mass cytometry panel to assess blood samples from multiple myeloma patients (Cohort 4; n=161)

| Antibody | Conjugate | Metal |
| --- | --- | --- |
| CD16 | 209Bi | Bismuth |
| CD24 | 161Dy | Dysprosium |
| CD11c | 162Dy | Dysprosium |
| CD57 | 163Dy | Dysprosium |
| CD45RO | 164Dy | Dysprosium |
| CD314 | 166Er | Erbium |
| CD38 | 167Er | Erbium |
| CD336 | 168Er | Erbium |
| HLA-DR | 170Er | Erbium |
| CD14 | 151Eu | Europium |
| CD56 | 155Gd | Gadolinium |
| CD158a | 156Gd | Gadolinium |
| CD27 | 158Gd | Gadolinium |
| CD28 | 160Gd | Gadolinium |
| CD159a | 165Ho | Holmium |
| CD8 | 115In | Indium |
| CD197 | 175Lu | Lutetium |
| CD19 | 142Nd | Neodymium |
| CD45RA | 143Nd | Neodymium |
| CD11b | 144Nd | Neodymium |
| CD4 | 145Nd | Neodymium |
| IgD | 146Nd | Neodymium |
| CD194 | 150Nd | Neodymium |
| CD335 | 141Pr | Praseodymium |
| FOXP3 | 147Sm | Samarium |
| PD-L1 | 148Sm | Samarium |
| Cd25 | 149Sm | Samarium |
| CD66b | 152Sm | Samarium |
| CD3 | 154Sm | Samarium |
| CD337 | 159Tb | Terbium |
| CD304 | 169Tm | Thulium |
| CD45 | 89Y | Yttrium |
| CD20 | 171Yb | Ytterbium |
| CD158b | 173Yb | Ytterbium |
| CD279 | 174Yb | Ytterbium |
| CD127 | 176Yb | Ytterbium |
